# Signaling pathway cloud regulation for *in silico* screening and ranking of the potential geroprotective drugs

**DOI:** 10.3389/fgene.2014.00049

**Published:** 2014-03-03

**Authors:** Alex Zhavoronkov, Anton A. Buzdin, Andrey V. Garazha, Nikolay M. Borisov, Alexey A. Moskalev

**Affiliations:** ^1^Department of Biological and Medical Physics, Moscow Institute of Physics and TechnologyDolgoprudny, Russia; ^2^First Oncology Research and Advisory CenterMoscow, Russia; ^3^The Biogerontology Research FoundationLondon, UK; ^4^Department of Experimental and Molecular Medicine, D. Rogachyov Federal Research Center of Pediatric Hematology, Oncology and ImmunologyMoscow, Russia; ^5^Shemyakin-Ovchinnikov Institute of Bioorganic Chemistry, Russian Academy of SciencesMoscow, Russia; ^6^Burnasyan Federal Medical Biophysical CenterMoscow, Russia; ^7^Department of Ecology, Syktyvkar State UniversitySyktyvkar, Russia; ^8^Laboratory of Molecular Radiobiology and Gerontology, Institute of Biology of Komi Science Center of Ural Branch of Russian Academy of SciencesSyktyvkar, Russia

**Keywords:** geroprotector, aging-suppressive drug, signaling pathway cloud, validation of drugs, transcriptomics

## Abstract

The major challenges of aging research include absence of the comprehensive set of aging biomarkers, the time it takes to evaluate the effects of various interventions on longevity in humans and the difficulty extrapolating the results from model organisms to humans. To address these challenges we propose the *in silico* method for screening and ranking the possible geroprotectors followed by the high-throughput *in vivo* and *in vitro* validation. The proposed method evaluates the changes in the collection of activated or suppressed signaling pathways involved in aging and longevity, termed signaling pathway cloud, constructed using the gene expression data and epigenetic profiles of young and old patients' tissues. The possible interventions are selected and rated according to their ability to regulate age-related changes and minimize differences in the signaling pathway cloud. While many algorithmic solutions to simulating the induction of the old into young metabolic profiles *in silico* are possible, this flexible and scalable approach may potentially be used to predict the efficacy of the many drugs that may extend human longevity before conducting pre-clinical work and expensive clinical trials.

The increasing burden of the aging on the economies of the developed countries is turning the quest to increase healthy life spans from an altruistic cause into a pressing economic priority required to maintain the current standards of living and facilitate economic growth (Zhavoronkov and Litovchenko, 2013). There is an urgent need to develop and validate interventions with geroprotective properties to increase the productive health spans of the working population and maintaining performance and avoiding loss of function (Kennedy, 2012).

While no doubt exists that aging is a complex multifactorial process with no single cause or treatment (Zhavoronkov and Cantor, 2011; Trindade et al., 2013), the issue whether aging can be classified as the disease is widely debated (Rattan, 2013). However, many strategies for extending organismal life spans have been proposed including replacing cells (Rodgerson and Harris, 2011) and organs, comprehensive strategies for repairing the accumulated damage, using hormetins to activate endogenous repair processes (Gems and Partridge, 2008; Gaman et al., 2011), modulating the aging processes through specific mutations, gene therapy (Bernardes De Jesus et al., 2012) and small molecule drugs (Kennedy and Pennypacker, 2013). An animal's survival strongly depends on its ability to maintain homeostasis and achieved through intracellular and intercellular communication within and among different tissues (Alcedo et al., 2013). Many strategies for the development and validation of drugs with geroprotective properties have been proposed to help maintain the homeostasis including drugs that act on specific targets or combinations of molecular pathways (Moskalev and Shaposhnikov, 2010, 2011; Zhavoronkov et al., 2012; Danilov et al., 2013) and epigenetic drugs (Vaiserman and Pasyukova, 2012). However, none of the proposed strategies for aging-suppressive drug development provide a roadmap for rapid screening, validation, and clinical deployment. No methods currently exist to predict the effects of currently available drugs on human longevity and health span in a timely manner. This is partly due to the absence of the clear panel of human processes involved in aging to effectively run clinical trials.

Many processes are involved in the aging of cells and organisms including telomere length (Lehmann et al., 2013), intracellular and extracellular aggregates, racemization of the amino acids and genetic instability. Both gene expression (Wolters and Schumacher, 2013) and DNA methylation profiles (Horvath et al., 2012; Horvath, 2013; Mendelsohn and Larrick, 2013) change during aging and may be used as biomarkers of aging. Many studies analyzing transcriptomes of biopsies in a variety of diseases indicated that age and sex of the patient had significant effects on gene expression (Chowers et al., 2003) and that there are noticeable changes in gene expression with age in mice (Weindruch et al., 2002; Park et al., 2009) resulting in development of mouse aging gene expression databases (Zahn et al., 2007) and in humans (Blalock et al., 2003; Welle et al., 2003; Park and Prolla, 2005; Hong et al., 2008; De Magalhaes et al., 2009).

Combination of protein-protein interaction and gene expression in both flies and humans demonstrated that aging is mainly associated with a small number of biological processes, might preferentially attack key regulatory nodes that are important for network stability (Xue et al., 2007).

Our prior work with gene expression and epigenetics of various solid tumors (Kuzmin et al., 2010; Mityaev et al., 2010; Zabolotneva et al., 2012a,b) using the OncoFinder system (www.oncofinder.com), provided clues that transcription profiles of cancer cells mapped onto the signaling pathways may be used to screen for and rate the targeted drugs that regulate pathways directly and indirectly related to aging and longevity. Instead of focusing on individual network elements, this approach involves creating the signaling pathway cloud, a collection of signaling pathways involved in aging and longevity each comprised of multiple network elements and evaluating the individual pathway activation strength. Despite significant advances in aging research, the knowledge of the aging processes is still poor, and combining all available factors involved in cellular aging, aging of the organisms, age-related diseases, stress-resistance, and stress-response along the many other factors into a comprehensive signaling pathway cloud may be more beneficial than focusing on the narrow collection of elements. The creation of the pathway cloud may allow for the annotated databases of molecules and other factors to be screened for effectiveness of individual compounds in replicating the “young” signaling activation profiles *in silico*.

Several new methods evaluating the robustness and response ability of the gene regulatory network have been developed and applied to gene expression data sets from young and old patients (Tu and Chen, 2013). Prior studies suggested that a combination of pathways, termed pathway cloud, instead of one element of the pathway or the whole pathway might be responsible for pathological changes in the cell (Voronkov and Krauss, 2013). Long-lived species like the sea urchin (*Strongylocentrotus franciscanus*) and naked mole rat (*Heterocephalus glaber*) that senesce at a slower rate than members of the same order show less transcriptome changes with age (Kim et al., 2011; Loram and Bodnar, 2012). Gene network analysis using gene expression data was effective in identifying the possible drug targets (Imoto et al., 2003; Savoie et al., 2003). *In silico* drug discovery algorithms that attempt to transform the metabolism to the healthy state have been proposed and validated (Yizhak et al., 2013). We theorize that in order to be effective, the geroprotector or a combination of aging-suppressive drugs must regulate the pathway cloud in a way that minimizes the difference in the net differences in pathway cloud activation or downregulation between samples of young and old patients. Small molecules and other factors that may influence gene expression may be ranked by their ability to minimize the net difference between the pathway activation profiles of young and old cells. The algorithms for calculating the ability of the potential geroprotector to minimize signaling disturbance may be parametric and account for the effects on specific targets within signaling pathways or machine learned.

Despite the differences in life span and aging phenotypes, many molecular mechanisms of aging are common in all eukaryotes. Pathway analysis revealed that there are many common age-related transcriptomic changes between different species, including yeasts, worms, flies, rodents, and human (Murphy et al., 2006). Hypothetically, the human orthologs of aging-related genes of model organisms are also involved in aging process. To select longevity-associated pathways for future analysis we perform the following procedure. Using (Tacutu et al., 2013) database we selected genes, where knockout, loss-of-function mutation, deletion or RNA interference significantly extended lifespan in several model organisms (yeasts *Schizosaccharomyces pombe* and *Saccharomyces cerevisiae*, nematode *Caenorhabditis elegans*, fruitfly *Drosophila melanogaster* and mouse *Mus musculus*) from 10 to 200%. We converted the obtained gene lists from different models to the general list of human orthologs where it is possible. 226 genes of 315 from our set were subjected to over-representation pathway analysis in (Kamburov et al., 2011). *P*-value and corrected by FDR *P*-value are calculated according to the hypergeometric test based on the number of physical entities present in both the predefined set of genes to each (Kanehisa et al., 2004) pathway and our list of aging-associated genes (Table 1). We established limits of 2 genes of minimum overlap with input list and 0.01 *p*-value cut off threshold.

**Table 1:** **KEGG pathways and list of aging-associated genes**.

**KEGG pathway name**	**Background quantity of genes in the pathway**	**Overlap with candidates list**	**%**	***p*-Value**	**FDR**
Citrate cycle (TCA cycle)	30	5	16.7	0.000401	0.0037
Ribosome	136	21	15.7	7.52E-13	6.21E-11
Parkinson‘s disease	131	20	15.4	3.87E-12	2.02E-10
Aldosterone-regulated sodium reabsorption	39	6	15.4	0.000165	0.00199
Type II diabetes mellitus	48	7	14.6	6.66E-05	0.00105
Oxidative phosphorylation	133	19	14.4	4.55E-11	1.78E-09
mTOR signaling pathway	60	8	13.3	3.89E-05	0.000764
Huntington‘s disease	183	24	13.2	7.92E-13	6.21E-11
Progesterone-mediated oocyte maturation	86	11	12.8	2.08E-06	4.66E-05
Insulin signaling pathway	142	17	12.1	8.01E-09	2.10E-07
Ovarian steroidogenesis	51	6	11.8	0.000735	0.00525
Long-term depression	60	7	11.7	0.000281	0.00294
Amyotrophic lateral sclerosis (ALS)	53	6	11.3	0.000905	0.00592
Alzheimer‘s disease	170	19	11.2	3.36E-09	1.05E-07
Cardiac muscle contraction	77	8	10.5	0.000214	0.0024
Gap junction	89	9	10.1	0.000118	0.00155
Prostate cancer	89	9	10.1	0.000118	0.00155
Estrogen signaling pathway	100	10	10.0	5.44E-05	0.00095
Colorectal cancer	62	6	9.7	0.00206	0.0101
Glioma	65	6	9.2	0.00263	0.012
Pancreatic cancer	66	6	9.1	0.00284	0.0124
GABAergic synapse	90	8	9.0	0.000631	0.00524
Adipocytokine signaling pathway	71	6	8.6	0.00382	0.0154
PPAR signaling pathway	71	6	8.5	0.0041	0.0157
Circadian entrainment	97	8	8.3	0.00104	0.00629
Prolactin signaling pathway	72	6	8.3	0.0044	0.0157
Chronic myeloid leukemia	73	6	8.2	0.0047	0.0164
Cholinergic synapse	113	9	8.0	0.000667	0.00524
Oocyte meiosis	112	9	8.0	0.000667	0.00524
Serotonergic synapse	114	9	8.0	0.000711	0.00525
Insulin secretion	87	7	8.0	0.00261	0.012
Gastric acid secretion	75	6	8.0	0.00537	0.0183
HIF-1 signaling pathway	106	8	7.5	0.00198	0.01
Peroxisome	81	6	7.5	0.00734	0.0226
TGF-beta signaling pathway	80	6	7.5	0.00734	0.0226
Cell cycle	124	9	7.3	0.00138	0.00803
Dopaminergic synapse	131	9	6.9	0.00192	0.01
Glutamatergic synapse	118	8	6.8	0.00366	0.0151
Proteoglycans in cancer	225	14	6.2	0.000348	0.00342
RNA transport	165	10	6.1	0.00268	0.012
Hepatitis B	147	9	6.1	0.00439	0.0157
HTLV-I infection	267	14	5.3	0.00161	0.00869
Chemokine signaling pathway	192	10	5.3	0.00732	0.0226
PI3K-Akt signaling pathway	347	16	4.6	0.00312	0.0133

As a result we revealed overrepresented cell signaling pathways (mTOR, insulin/IGF-1, PI3K-Akt, PPAR, HIF-1, TGF-beta, chemokines, adipocytokine, prolactin, estrogen), general metabolism (TCA cycle, ribosome, oxidative phosphorylation), RNA transport, cell cycle and meiosis, gap junction, peroxisome, cyrcadian rhythm, different synapse types (dopaminergic, glutamatergic, cholinergic, serotonergic, GABAergic), gastric acid secretion as well as age-related diseases pathways (Parkinson's disease, type II diabetes mellitus, Huntington's disease, long-term depression, amyotrophic lateral sclerosis, Alzheimer's disease), Hepatitis B, HTLV-I infection and cancer pathways (prostate cancer, colorectal cancer, glioma, pancreatic cancer, chronic myeloid leukemia, proteoglycans in cancer). We considered obtained such a way pathways as probably associated with the human longevity. Human genes known as key activators/repressors of these pathways may be used in provided further mathematical model.

The methods that may be applied for the possible analysis of geroprotector efficiency by pathways regulation have been arisen from our research experience of cell signaling pathways. As far as we have seen before (Kiyatkin et al., 2006; Borisov et al., 2009; Kuzmina and Borisov, 2011), most signal transduction proteins are essentially far from saturation even at the peak concentrations of the activated form in comparison with the total protein abundances. Thus, we can consider that all activator/repressor genes/proteins have equal importance for the pathway activation/downregulation, and then arrive at the following assessment function for the overall signal pathway cloud disturbance outcome (*SPCD*) is proportional to the following estimator function,

$$SPCD=\frac{\prod_{i\;=\;1}^{N}{\left[AGEL\right]}_{i}}{\prod_{j\;=\;1}^{M}{\left[RGEL\right]}_{j}}$$

Here the multiplication is done over all possible activator and repressor proteins in the pathway, and [*AGEL*]_*i*_ and [*RGEL*]_*j*_ are gene expression levels of an activator *i* and repressor *j*, respectively. To obtain an additive rather than multiplicative value, it is enough just turn from the absolute values of the expression levels to their logarithms, arriving at the *pathway activation strength* (*PAS*) value for each pathway (see Figure 1). To obtain the values of *Old (case)-to-Young ratio*, *YOR*_*n*_, one just has to divide the expression levels for a gene *n* in the sample taken for the senescent person by the same average value for the normalized young group. The discrete value of *ARR* (*activator*/*repressor role*) equals to the following numbers:

**Figure 1:**
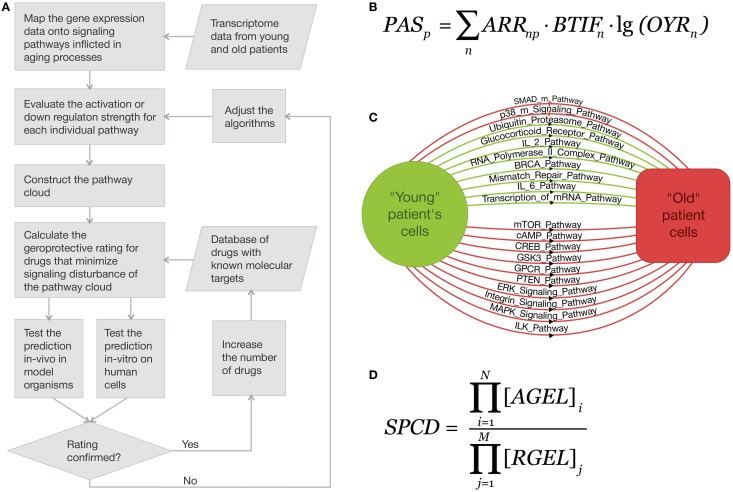
**Gene expression-based approach to *in silico* screening for drugs with geroprotective properties and estimating the predicted efficacy. (A)** Using signaling pathway cloud regulation for theoretical *in silico* aging-suppressive drug identification and ranking. The proposed method for identifying and ranking of geroprotective drugs by evaluating the net effect on the many elements of signaling pathway cloud that brings the “old” metabolic state closer to the “young.” **(B)** An example of how multiple pathways are activated and down-regulated during aging. **(C)** Pathway Activation Strength (PAS) is the logarithmic additive value that characterizes the up-/downregulation of signaling pathways. **(D)** Function for the overall signal pathway cloud disturbance outcome (SPCD).

−1, when the gene/protein *n* is a repressor of pathway excitation;

1, if the gene/protein *n* is an activator of pathway excitation;

0, when the gene/protein *n* can be both an activator and a repressor of signal transduction;

0.5 and −0.5, respectively, if the gene/protein *n* is more an activator or repressor of the signaling pathway *p*.

The information about the activator/repressor role of a particular gene/protein may be obtained from the analysis of open-access or customized pathway databases and from the literature.

The Boolean flag of *BTIF* (*beyond tolerance interval flag*) equals to zero when the *OYR* value lies within the tolerance limit, and to one when otherwise. During the current study, we have admitted that the *OYR* lies beyond the tolerance limit if it satisfies simultaneously the two criteria. First, it either higher than 3/2 or lower than 2/3, and, second, the expression level for a corresponding gene from an old patient of an individual patient differs by more than two standard deviations from the average expression level for the same gene from a set of analogous young tissue/organ samples.

We propose a new computational approach for identifying and rating the variety of factors including small molecules, peptides, stress factors and conditions with the known effects on the transcriptomes at different ages of one or more cell or tissue types or known targets (Figure 1A). The approach may be used for general geroprotector screening, but after the validation of the algorithms *in vivo* and *in vitro* may be expanded to identify and predict the efficacy of personalized aging-suppressive intervention regimens for individual patients based on the transcriptome information from various tissue biopsies and blood samples.

The generic geroprotector rating approach involves collecting the transcriptome data sets from young and old patients and normalizing the data for each cell and tissue type, evaluating the pathway activation strength (PAS) for each individual pathway (Figure 1B) and constructing the pathway cloud (PC, Figure 1C) and screen for drugs or combinations that minimize the signaling pathway cloud disturbance (SPCD, Figure 1D) by acting on one or multiple elements of the pathway cloud. Drugs and combinations may be rated by their ability to compensate the changes in signaling pathway activation patterns that are related to aging, thus bringing all the *PAS* values for the general set of the pathways as close to zero as possible. Since many of the drugs approved for use in humans have known molecular targets and some have been screened for the impact on longevity in model organisms (Ye et al., 2013), the predictions may be then tested both *in vitro* and *in vivo* on human cells and on model organisms such as rodents, nematodes and flies to validate the screening and rating algorithms.

## Conclusion

Longevity studies of aging-suppressive drug efficiency in higher mammals take several years and decades and may cost millions of dollars. An intelligent process for predicting the activity and ranking the geroprotective activity of various factors and strengthening the prediction in rapid and cost-effective studies on cell cultures and model organisms may help increase the longevity dividend of these studies. In this paper we propose a method for *in silico* screening and ranking of drugs and other factors that act on many signaling pathways implicated in aging processes by calculating their ability to minimize the difference between signaling pathway activation patterns in cells of young and old patients and confirming the results using *in vivo* and *in vitro* studies.

### Conflict of interest statement

The authors declare that the research was conducted in the absence of any commercial or financial relationships that could be construed as a potential conflict of interest.
